# MRGPRX2 and Adverse Drug Reactions

**DOI:** 10.3389/fimmu.2021.676354

**Published:** 2021-08-06

**Authors:** Benjamin D. McNeil

**Affiliations:** Division of Allergy and Immunology, Feinberg School of Medicine, Northwestern University, Chicago, IL, United States

**Keywords:** MRGPRX2 receptor, anaphylaxis, mast cells, perioperative anaphylaxis, morphine, atracurium, vancomycin, rocuronium

## Abstract

Many adverse reactions to therapeutic drugs appear to be allergic in nature, and are thought to be triggered by patient-specific Immunoglobulin E (IgE) antibodies that recognize the drug molecules and form complexes with them that activate mast cells. However, in recent years another mechanism has been proposed, in which some drugs closely associated with allergic-type events can bypass the antibody-mediated pathway and trigger mast cell degranulation directly by activating a mast cell-specific receptor called Mas-related G protein-coupled receptor X2 (MRGPRX2). This would result in symptoms similar to IgE-mediated events, but would not require immune priming. This review will cover the frequency, severity, and dose-responsiveness of allergic-type events for several drugs shown to have MRGPRX2 agonist activity. Surprisingly, the analysis shows that mild-to-moderate events are far more common than currently appreciated. A comparison with plasma drug levels suggests that MRGPRX2 mediates many of these mild-to-moderate events. For some of these drugs, then, MRGPRX2 activation may be considered a regular and predictable feature after administration of high doses.

## Introduction

Acute adverse reactions to therapeutic drugs are those which occur within minutes to hours of drug exposure, and many of these present clinically as allergic episodes (1, 2). Mild-to-moderate symptoms include rash, erythema, pruritus, tachycardia, local tissue swelling, moderate bronchospasm, transient hypotension, and gastrointestinal distress (3, 4). The most extreme of these reactions are classified as “anaphylaxis” and can be life-threatening; these include more severe hypotension, bronchospasm, and tissue swelling, and even collapse of the cardiovascular system (3, 4).

Most of these are assumed to be driven by activation of mast cells by drug-specific Immunoglobulin E (IgE) antibodies, which are called Type I immediate hypersensitivity reactions (1, 2, 5). Prior exposure to the drug, or to a compound with a structurally similar element, stimulates production of antibodies that recognize the drug or a conjugate formed when the drug or a metabolite binds to an endogenous protein. These antibodies then associate with high-affinity IgE receptors on the surface of mast cells in a manner that leaves their drug-binding sites free. When a drug recognized by the antibodies is administered, it (or the conjugate) binds to multiple antibodies at the same time. This brings the IgE receptors associated with the antibodies into prolonged close contact, triggering activation of the receptors and the release of mediators like histamine that generate the allergic responses (6).

Another cause of acute mast cell activation has been proposed, in which drugs trigger reactions very similar to Type I events – but without the need for antibodies or immune priming – by activating mast cells directly through a receptor called Mas-related G protein-coupled receptor X2 (MRGPRX2). MRGPRX2 is a seven transmembrane G protein-coupled receptor which is expressed almost exclusively by a subset of mast cells that populate connective tissues like the skin (7, 8). It is classified as an orphan receptor (9), meaning that the ligand(s) it is intended to recognize has not been determined. However, multiple screens with hundreds of small molecules, peptides, and proteins have established that it is responsive to a wide range of molecules, and that the overwhelming majority of them carry a net cationic, or positive, charge (8, 10–14). A recent review identified that most also have bulky hydrophobic groups, perhaps to increase affinity for plasma membranes (15). In 2015 a study reported that several therapeutic drugs with cationic groups, all of which induce high rates of allergic-type reactions, are agonists for MRGPRX2 (12). Moreover, activation of a cell line called LAD2, which has properties similar to human mast cells and often is used as a surrogate because primary cells are very difficult to extract, was dependent upon MRGPRX2 (12). Other drugs capable of activating MRGPRX2 have since been found, many of which also trigger allergic-type events. This finding raises the possibility that side effects that appear to be Type I – i.e., allergic and IgE-mediated – may in some cases arise instead from direct activation of mast cells through MRGPRX2. Such events have been called “pseudo-allergic” or “anaphylactoid” to distinguish them from true allergies. All events that present as allergic episodes will be referred to as “allergic-type” in this review, as the etiology is not always clear.

This review will present an analysis of the frequencies of allergic-type events for many drugs/MRGPRX2 agonists that are particularly closely associated with such events. Calculated EC_50_ values for MRGPRX2, compiled from several studies, are presented in **Table 1** (12–14, 16–19). Two specific issues are addressed for each drug: 1.) whether the mild-to-moderate events truly are mast cell-mediated; and 2.) whether MRGRPX2 involvement is supported. The first issue is important because, while anaphylaxis elicits a stereotyped and coordinated set of symptoms with a clear mast cell origin, the milder events only include some of these, and mast cell activation is not the only possible cause of the symptoms. The second issue, of whether MRGPRX2 is involved, is impossible to prove without specific antagonists. However, if events are much more common only when plasma levels are high enough to activate MRGPRX2, it certainly supports a role for this receptor. Therefore, plasma concentrations are provided for each drug. A more detailed discussion on methods of distinguishing IgE from MRGPRX2 or other non-IgE origins is provided in Section II.

**Table 1:** Calculated EC50 values for selected MRGPRX2 agonists.

Name	EC_50_	Reference
Vancomycin	~ 60 micrograms/ml	(14)
Atracurium	28.6 micrograms/ml	(12)
Mivacurium	39 micrograms/ml	(16)
Cisatracurium	103 micrograms/ml	(17)
Rocuronium	261 micrograms/ml	(12)
Morphine	4.5 - 7 micromolar(1.3 – 2.0 micrograms/ml)	(13, 18, 19)
Ciprofloxacin	6.8 micrograms/ml	(12)
Levofloxacin	22.7 micrograms/ml	(12)
Moxifloxacin	9.9 micrograms/ml	(12)

The most surprising finding from this analysis is that mild-to-moderate allergic-type events can be very frequent, much more so than generally presumed. These events generally are neglected in favor of anaphylactic episodes, which are much more serious but are extremely rare. In contrast, mild-to-moderate events have been reported to occur in a majority of patients at some drug dosages. These are not trivial and may have serious impacts on health when patients already are highly compromised. Peak drug plasma concentrations support MRGPRX2 involvement in these events for several drugs; this suggests that MRGPRX2 activation might be considered a common, not a rare, feature when these drugs are administered.

## Determination of IgE- *vs.* MRGPRX2-Mediated Mast Cell Activation

A pressing issue in the field is how to determine whether mast cell activation is mediated by IgE or MRGPRX2 when a patient has suffered an allergic-type event due to a drug that is an MRGPRX2 agonist. These drugs also may be immunogenic, so simply exhibiting MRGPRX2 agonism does not rule out IgE. Technically, distinguishing between these is not yet possible because there are no biomarkers that reliably identify or exclude one or the other mechanism, such as a mediator only released after stimulation of one but not the other receptor. However, specific measurements can be made that support the involvement of each pathway.

### MRGPRX2 Involvement

MRGPRX2 should be suspected if an event is *only* observed at concentrations high enough to activate the receptor, and resolves when the concentration drops below this. As described in detail in th next section, the drug concentrations needed to activate MRGPRX2 are very high and only achieved transiently for most drugs. Many allergic-type reactions also are very transient and only occur at very high drug concentrations. In contrast, there is a widespread assumption that IgE-mediated mast cell activation occurs even at very low concentrations of an antigen – for example, food allergies only require miniscule amounts of food- though this is not proven for every allergy. Another factor is that mediator release after IgE-driven mast cell activation persists for much longer than after antibody-independent mast cell stimulation (20), so events that are short-lived are less likely to be IgE-driven, especially if they only occur at high drug concentrations and disappear when plasma or tissue concentrations drop below EC_50_ values for MRGPRX2.

EC_50_ values for MRGPRX2 can be used to determine whether plasma or tissue drug concentrations are high enough to activate the receptor. However, several additional factors should be considered when evaluating these. First, plasma concentration measurements may not reflect concentrations in some tissues; specific examples are discussed in the fluoroquinolones and neuromuscular blocking drug sections. Second, EC_50_ values must be taken in context, as caveats exist. The values were calculated for the most common MRGPRX2 variant, but dozens of others with slightly different amino acid compositions, due to natural variations in the coding DNA, have been identified (21, 22). These sometimes have altered properties; most of the ones characterized are loss-of-function, but ones with enhanced signaling have also been reported (23, 24). It is quite possible that alleles that respond to much lower drug concentrations are expressed by some patients, and if so, EC_50_ values for those variants should be used instead. Also, MRGPRX2 expression levels vary tremendously between subjects (25), and those with abnormally high expression may also respond to low drug concentrations, even when the canonical receptor variant is expressed. Another consideration is that EC_50_ values usually are calculated in cell lines, not in primary cells. Finally, concurrent illnesses may either enhance or reduce mast cell responsiveness, or how tissues respond to mast cell mediators. An example is provided below in the vancomycin section, in which bacterial infections appear to dramatically reduce systemic mast cell responses. On the other hand, patients with chronic spontaneous urticaria appear to have much stronger responses to MRGPRX2 agonists (26). This is an emerging topic and more research needs to be performed, but it is clear that comorbidities can have a profound influence on allergic-type reactions.

### IgE Involvement

IgE-mediated mast cell activation should be suspected when events occur at low drug concentrations, when the events are of long duration, or when drug-specific IgE titers are high. Four tests are recognized by the World Allergy Organization to help determine whether a patient has drug-specific IgE antibodies: a skin prick test, intradermal injection of the drug, plasma or serum IgE quantification, and basophil activation tests (27). Protocols are not standardized, and interpretation of the results can be quite controversial (28, 29). Of these, skin prick and intradermal injection tests are, by far, the most common methods for identifying IgE involvement. The concept behind them is that concentrations are too low to activate MRGPRX2, but are sufficient to trigger IgE reactions. However, concentrations are not standardized and in many cases likely are enough to activate MRGPRX2 – for instance, a commonly-used skin prick test concentration for morphine and atracurium is 1 mg/ml (30), dozens of times higher than their EC_50_ values for MRGPRX2 activation (**Table 1**). Intradermal concentrations generally do not exceed these values, though morphine is recommended at 10 micrograms/ml, over 5 times higher than its EC_50_ value. As mentioned above, MRGPRX2 variants may have greater sensitivity and may trigger signaling at even lower drug concentrations. Even when several tests are used, the results can be equivocal. In one study, all four tests were conducted in each of 140 instances of anaphylaxis after administration of the neuromuscular blocker rocuronium (31). Strikingly, the tests all were in agreement in less than 15% of the cases. This is not meant to imply that IgE tests are not useful, only that they are not yet optimized, and that tests for MRGPRX2 involvement should be conducted, as well.

Unfortunately, the assays described for MRGPRX2 and IgE essentially never are conducted together. Plasma drug concentrations and MRGPRX2 allele analysis are almost exclusively limited to controlled experiments in which mild-to-moderate but not anaphylactic events are observed. Conversely, IgE tests are used as a diagnostic only after anaphylactic episodes. Until they are all performed in tandem, even a perfect IgE test cannot rule out MRGPRX2; likewise, evidence of extraordinarily high drug concentrations cannot rule out IgE. It even seems quite plausible that both can operate together in some cases. It is hoped that future studies with a more comprehensive approach will be undertaken to help clarify this matter, particularly in cases of anaphylaxis.

## Allergic-Type Adverse Event Frequencies and Analysis

This section summarizes and analyzes the available data on allergic-type event frequency for several drugs known to have MRGPRX2 agonist properties. It also discusses evidence for and against a mast cell origin for these events, as well as peak plasma drug concentrations to help evaluate whether MRGPRX2 plays a role when mast cells are involved. Plasma drug concentrations are almost totally unknown in patients who have suffered anaphylactic episodes, so correlations cannot be made for these events.

### Vancomycin

Vancomycin is a glycopeptide antibiotic used for difficult-to-treat Gram-positive bacterial infections like methicillin-resistant *Staphylococcus aureus* (32, 33). It is given orally or, more frequently, intravenously as a slow infusion, and is closely associated with allergic-type reactions – often regrettably described as “Red Man Syndrome” – that begin during or shortly after infusion (34).

#### Allergic-Type Event Frequency and Mast Cell Dependence

Allergic-type reactions are the most common side effects of vancomycin, and are characterized by erythema of the head and neck, hypotension, tachyphylaxis, pruritus, and occasional angioedema (33, 35, 36). These usually are associated with elevated plasma histamine (37–41), and often can be mitigated by antihistamines (42–46), confirming mast cell involvement in these reactions.

The reported frequency of allergic-type reactions is highly variable, with most studies reporting either 5% or less [e.g (47–54)] or over 70% [e.g (38–40, 42, 46, 49, 55–57)]. No systematic differences in dosage exist between the high and low incidence studies, suggesting that other factors were responsible for this vast disparity. Notably, with very rare exceptions (43, 58), the studies reporting high incidence rates were conducted specifically to examine side effects, while the studies with low rates were designed to assess antibacterial efficacy. It is plausible that the different aims resulted in different thresholds for what constituted a medically relevant side effect.

Another difference between the high and low incidence studies is the makeup of the study populations. The high incidence studies examined healthy subjects or hospital patients without infections, while the low incidence studies were almost all of patients with severe bacterial infections. Mast cells can be activated by bacteria (59), and it is possible that persistent activation during infection leads to mast cell desensitization to further stimuli, and/or systemic desensitization to mast cell mediators. In support of this, one small study compared responses in healthy volunteers to those in infected patients, and found that no infected patients had any reactions, while nearly all of the healthy controls did (49). No definitive conclusion can be drawn yet, but the inverse correlation between infection and mast cell responsiveness appears to be quite strong.

In a massive study of over four million patients given vancomycin, anaphylaxis was reported to occur with a frequency of 0.018%, or approximately 1 in 5000 (54).

#### Peak Plasma Concentrations and Potential MRGPRX2 Involvement

Vancomycin is a weak agonist, with a calculated EC_50_ of about 60 micrograms/ml (14). Recorded peak plasma levels of vancomycin cluster around the 30-50 micrograms/ml range, which is enough to activate mast cells but not to a large extent. Patients with more severe reactions may have plasma levels on the upper end of this range – indeed, levels exceeding 70 micrograms/ml have been reported (55). Importantly, most measurements were taken after the infusion was complete and they may underreport the actual peak. Small differences in concentration are important when considering MRGPRX2 activation, as the reported dose-response curve in cell lines is very steep (14) and slight changes can have large effects. For example, in one study, reducing the average peak concentration from 65.7 to 40.3 micrograms/ml was sufficient to completely abolish all allergic-type reactions (55). Plasma drug levels and MRGPRX2 allele expression were not recorded in the large study that calculated anaphylaxis rates (54), so no correlations are available.

### Atracurium, Cisatracurium, and Mivacurium

These all are non-depolarizing neuromuscular blocking drugs (NMBDs). NMBDs are routinely used during surgical procedures to facilitate tracheal intubation of breathing tubes, and to reduce aberrant muscle activity during the surgeries. They bind to and block acetylcholine receptors expressed by muscles, preventing innervation by nerves (60). High doses of atracurium and mivacurium are associated with allergic-type side effects (61); these are much less frequent after cisatracurium administration (62, 63), which may be due to the fact that relatively low doses are used.

#### Allergic-Type Event Frequency and Mast Cell Dependence

Non-depolarizing NMBDs are associated with flushing, erythema, and hypotension (61). Preclinical studies suggested that high doses of atracurium would cause hypotension and histamine release in patients (64). This was indeed the case – after rapid injection of 0.5 mg/kg or more, elevated histamine levels in the plasma were recorded (65–69), drops in mean arterial blood pressure (MAP) of at least 20% were observed in most studies, and this could be blocked by pre-treatment with a combination of H1 and H2 histamine receptor antagonists (65–68). Flushing or erythema also were blocked in studies that monitored this AE (66, 69). The choice of antihistamine may be important, as some can counteract their own effects by blocking the enzyme that breaks down histamine, which would elevate histamine levels (65). One study demonstrated that the anesthetic thiopental, commonly used with atracurium, also can cause a drop in MAP, and suggested that this is the primary reason for the drop (69). However, this does not explain the cases where thiopental was not used (68) or was administered well before atracurium (66, 67), nor does it explain why the drop in MAP could be abolished by slowing down the atracurium injection time (65, 66), which would produce a lower peak plasma concentration. Taken together, the data strongly suggest that the immediate drop in MAP and cutaneous allergic-type effects, the primary side effects of atracurium, are caused by mast cell activation.

Mivacurium injection is associated with elevated plasma histamine levels, flushing/erythema, and drops in mean arterial pressure (MAP) of greater than 20%, which all correlate with the speed of injection and drug dose. Elevated histamine levels were frequently observed when measured, sometimes in more than 50% of patients (70–73). Flushing/erythema has been observed in from 6% to 73% of patients (70–72, 74–82). MAP changes also occur at a lesser frequency, from 0% to 50% (70, 72, 73, 77, 80–83). Studies that examined all at once found that MAP changes nearly always were accompanied by flushing and histamine release (70, 73, 78). Antihistamines block all these effects (72, 78). It should be noted that occasional studies only reported average changes in histamine levels and/or mean arterial pressure, which sometimes did not achieve significance as a group (71, 79, 82, 84). This does not mean that zero patients in the group suffered from an AE. The heterogeneity of responses, compared to atracurium, likely stems from the variety of mivacurium doses and speeds of injection.

Cisatracurium is very rarely associated with allergic-type side effects, with incidence rates of 0.5% or less (62, 63), even in patients with existing cardiovascular morbidity (85). Elevated plasma histamine levels, though usually mild, have been recorded after high doses (0.2 mg/kg or more) of cisatracurium in approximately 5-10% of patients, so it is possible that the allergic-type events are indeed mast cell-mediated (86–89).

Recent studies have estimated the incidence of atracurium-induced anaphylaxis at approximately 1 in 20,000-50,000 (90–93). Anaphylactic episodes after cisatracurium administration are exceedingly rare, as low as 1 in 250,000 (92), though data are not common and estimates are not necessarily representative. Anaphylaxis after mivacurium was estimated as occurring in less than 1 in 1,000,000 administrations in a recent study (90), though, as with cisatracurium, data are relatively sparse, compared to rocuronium and atracurium. Plasma drug levels and MRGPRX2 allele data are not available for these events.

#### Peak Plasma Concentrations and Potential MRGPRX2 Involvement

Peak recorded plasma concentrations after rapid atracurium injections are usually less than 10 microgram/ml range, even when high concentrations of 0.5 mg/ml or more are administered (94, 95). This is lower than the calculated EC_50_ for MRGPRX2 of 28.6 micrograms/ml (12), and it is not clear from intradermal injection studies that mast cells respond to lower concentrations (96). However, even though measurements were taken 1-2 minutes after injection, there is reason to believe that these are not truly peak plasma or interstitial concentrations. Two studies found that extending injection times from 5-30 seconds to 75 seconds was sufficient to abolish most AEs (65, 66). This suggests that rapid injections produce plasma or interstitial concentrations somewhere in the body that are enough to activate mast cells; these are achieved for only several seconds and a very slight reduction in injection speed is enough to prevent this. These might not be captured in blood samples, even shortly after injection; notably, the cited pharmacokinetic studies did not include information about injection speed.

Peak reported plasma concentrations of the combined isomers of mivacurium cluster in the 3 to 10 micrograms/ml range when measurements are taken within a few minutes of injection (97–101). These also are too low for efficient activation of MRGPRX2, if the recorded EC_50_ of 39.0 micrograms/ml is accurate (16), and skin tests provide little evidence for activation at lower concentrations. Interestingly, 10-15 second injection times produced far more AEs than when administration was 30 seconds or more (70, 77, 80). This suggests that, as with atracurium, the true peak plasma and/or interstitial concentrations may be missed by the recordings.

Data on peak cisatracurium plasma concentrations are relatively scarce, but usually are less than 2 micrograms/ml (102, 103). This is far below the calculated EC_50_ of 103 micrograms/ml (17). These seem to be too far apart to consider any side effects to be MRGPRX2-related. However, skin reactions to intradermal cisatracurium have been observed at 12 micrograms/ml (104, 105), and LAD2 cell activation by cisatracurium is dependent on MRGPRX2 (17), so it is possible that the EC_50_ for primary mast cells may be lower.

### Rocuronium

Rocuronium is a member of the aminosteroid group of NMBDs that, like atracurium, acts as a muscle nicotinic receptor antagonist (60). Its onset is only slightly slower than the fastest-acting NMBD, succinylcholine, while its duration is longer (106). One attractive reason to use rocuronium is that its effects can be reversed rapidly by administering sugammadex, which binds to and inactivates rocuronium (107) and allows for precise control over paralysis. Unlike atracurium, very few adverse events related to off-target activity like mast cell degranulation are reported (106, 108–111). Somewhat surprisingly, the incidence of anaphylactic reactions, while still rare, has been estimated in some studies as being higher after administration of rocuronium that of most other NMBDs (91, 93).

#### Allergic-Type Event Frequency and Mast Cell Dependence

The most commonly-reported acute side effect specifically associated with rocuronium is tachycardia (112). Tachycardia, or increase in heart rate, has been observed with histamine-releasing drugs, but in the case of rocuronium, this is thought to be caused by off-target block of acetycholine receptors that regulate cardiac pacemaker activity (112). In fact, elevated histamine levels are extremely rare (72, 73, 113) and immediate hypersensitivity events are not observed in the vast majority of patients (108–111), though occasional mild skin reactions do occur (72). Estimates of anaphylaxis are highly variable (90, 114, 115) but are as high as 1 in 2500-4000 patients (91, 92, 116).

One plausible mast cell-related AE is an injection site reaction, which occurs in up to 80% of patients given rocuronium (106). However, while intravenous rocuronium can cause a local rash (117), intravenous and intradermal rocuronium injections are associated not with itch – typical of mast cell-driven reactions – but with sharp pain and involuntary limb withdrawal (118, 119). One study in mice suggests that rocuronium directly activates skin C-fibers, which transmit noxious sensations like pain (119). This apparently is pH-dependent, as neutralizing its pH from 3.5 to 7.4 abolished pain sensation in one human study (120). Another study reported that pain was reduced after pretreatment with the antihistamine chlorpheniramine maleate (121). However, it is quite possible that this is due to off-target activity, as the dosage given was 10 times higher than the standard amount and also was given as an intravenous bolus, which results in extremely high plasma concentrations, compared to an equivalent oral dose (122). In sum, mast cells may play a role in injection site reactions, but it seems likely that other mechanisms also contribute.

#### Peak Plasma Concentrations and Potential MRGPRX2 Involvement

Peak recorded plasma concentrations of rocuronium within a few minutes after rapid injection are between 6-15 micrograms/ml (123–128). The calculated EC_50_ for MRGRPX2 activation is 261 micrograms/ml (12), so even with the caveat that plasma concentration measurements underestimate the peak when taken after infusion, it seems impossible that MRGPRX2 could be systemically activated. This readily explains why histamine-associated AEs are so rare. MRGPRX2 alleles and drug concentrations are generally not available for patients who have suffered anaphylactic episodes. One study identified expression of an allele in one patient (129), though this does not appear to increase receptor sensitivity (130).

Injection site reactions may involve MRGPRX2. Rocuronium is supplied as a 10 mg/ml injection solution, which is much higher than the reported EC_50_ for MRGPRX2 of 261 micrograms/ml (12), and the threshold for evoking wheal and flare after intradermal injection is as low as 61 micrograms/ml (104). Local leakage of the drug into the area surrounding the injection site may trigger MRGPRX2-mediated mast cell activation.

### Morphine

Morphine is a small molecule alkaloid which is used clinically to activate endogenous opioid receptors and relieve pain. It usually is administered orally, intravenously, or spinally as an epidural or intrathecal injection. It has been linked to allergic-type reactions for over 100 years (131).

#### Allergic-Type Event Frequency and Mast Cell Dependence

Pruritus is one of the most commonly-reported adverse events of any kind associated with morphine (132–134). Frequencies after epidural administration are from 8.5% to over 50% (135–137); 30% - 100% after intrathecal administration (136–138); up to 40% after intravenous administration (139); and generally 2 - 10% after oral delivery (140), though higher rates have been reported (141). Oddly, other mast cell-associated adverse events like rash and hypotension are not nearly as common, and are rarely mentioned in clinical studies that use typical drug dosing regimens (132–134). The unusually specific dominance of pruritus can be explained, at least in part, by the fact that morphine and other opioid receptor agonists can engage a mast cell-independent mechanism to trigger pruritus. The details of this mechanism have not been fully worked out, but some evidence suggests that it is mediated by a subset of opioid receptor-expressing neurons in the spinal cord that specifically mediate itch transmission (142). This may be why epidural and intrathecal administration of morphine still trigger pruritus – in fact, they have the highest incidence rates of all routes of administration – even though they bypass systemic exposure and do not activate skin mast cells at all. This also may explain why the mu opioid receptor agonist fentanyl, which does not activate mast cells (or MRGPRX2) (13, 143), induces pruritus with incidence rates comparable to, though somewhat less than, morphine (144, 145). Thus, it is not clear how much of a role mast cells play in morphine-induced pruritus after normal clinical doses.

High doses of intravenous morphine produce side effects like flushing, changes in mean arterial pressure and lowered vascular resistance (131, 146–150), which almost certainly are mediated by mast cells. Plasma histamine levels usually were highly elevated when measured (146, 147, 150, 151), and in two studies the cardiovascular effects were reduced by pretreatment with H1 and H2 receptor antagonists (147) or the H1 receptor antagonist promethazine (149). Local administration of high doses of morphine into the forearm by skin prick, intradermal injection, or infusion into a local artery produced wheals and blood vessel dilation (often reported as a “flare”), which are common markers of mast cell activation and which could be reduced by antihistamines (152–156), though it is important to note that not all of the drugs used are specific for histamine receptors. Interestingly, an *in vitro* study of rat aortic endothelial cells demonstrated that morphine could influence their behavior directly through opioid receptors (157), suggesting a direct effect of morphine on blood vessels. This may have a minor role in humans, as well, as skin responses could partially be blocked by the opioid receptor antagonist naloxone (154, 156), at least when relatively low morphine doses were used (158). Still, taken together, the studies strongly suggest that most of the vascular changes induced by high doses of morphine are due to mast cell activation.

Anaphylaxis after morphine administration is thought to be exceedingly rare, though exact calculations are lacking. It has been proposed that some deaths from overdoses may involve anaphylaxis, but this is still unclear (159).

#### Peak Plasma and Tissue Concentrations, and Potential MRGPRX2 Involvement

Injection site reactions after morphine administration likely are due to MRGPRX2, as formulations usually are at 10 mM concentrations or higher and are well above the EC_50_ for MRGPRX2 activation of 4.5 to 7 µM (13, 18, 19). Typical systemic doses of morphine do not achieve plasma concentrations high enough to activate MRGPRX2 to a significant extent. For instance, peak concentrations rarely exceed 14 nM after oral dosing (160) and usually are 2 µM or less, often substantially so, after intravenous administration (161–166). This lends more support to a mast cell-independent origin for morphine-induced pruritus. Higher doses and/or those delivered rapidly are much more likely to result in concentrations that exceed the EC_50_, though not enough recordings have been made to determine just how high these are. As described above, this is when typical events like rash and swelling are seen. Since human skin mast cells do not express opioid receptors (7), it appears likely that most true mast cell-mediated events are mediated by MRGPRX2.

### Fluoroquinolones

Fluoroquinolones are a group of small molecule antibiotics which are structurally similar and are effective against Gram-positive and Gram-negative bacteria (167). Popular members are ciprofloxacin, levofloxacin, and moxifloxacin. They are administered orally or intravenously. Fluoroquinolones are associated with a constellation of mild-to-moderate adverse events, including typical allergic-type effects and others that potentially have a mast cell component. Fluoroquinolones also are linked to extremely serious side effects (168–172), which, while rare, are common enough that the FDA and European Medicines Agency now discourage their use for relatively mild infections, as the risks might outweigh the benefits (173). One of these, anaphylaxis, certainly is related to mast cells; of the others, tendinopathy and tendon rupture have been linked to mast cells in other diseases.

#### Allergic-Type Event Frequency and Mast Cell Dependence

Fluoroquinolones have a broad side effect profile and are not as clearly linked to mast cell activation as the other drugs in this review. Surprisingly, no systematic human studies have been carried out – for instance, measuring blood histamine or tryptase levels, and pretreating patients with antihistamines – to assess which of the symptoms are mast cell-driven. However, many frequently reported mild-to-moderate adverse events are highly suggestive of allergic-type reactions. Rash, pruritus, injection site reactions, and hypotension have been reported in 1-4% of patients (174–177), though occasionally frequencies are much higher (178). A relatively generic description of “allergy” occasionally is reported, with frequencies of up to 2% (179). Gastrointestinal symptoms, which could be driven by mast cells (180), occur at frequencies of up to 20% (170). With the exception of anaphylaxis, the severe side effects of fluoroquinolones – tendinitis and tendon rupture, peripheral neuropathy, central nervous system effects, increased risks of damage to the aorta, and decreases in blood sugar (171) – are not typically linked to mast cell activation. Interestingly, mast cells have been proposed to influence tendon healing after injury, perhaps weakening them (181, 182), so it is possible that they are involved in some way in fluoroquinolone-induced tendon inflammation. The risk of tendinopathy or tendon rupture depends dramatically on many other factors, including activity level, age, and use of corticosteroids, but fluoroquinolone use can increase this by several-fold (183). Unfortunately, the link between mast cells and any of the above symptoms remains speculative; an understanding of the full extent of mast cell involvement in fluoroquinolone-induced AEs awaits dedicated studies of the subject.

Anaphylaxis is rare but increasing in frequency; in fact, fluoroquinolones now are the second-most frequent cause of drug-induced anaphylaxis in total cases, behind beta-lactams (172). The calculated frequency of these events is as high as 1 in 20,000 administered doses, though other estimates are lower (184).

#### Peak Plasma and Tissue Concentrations, and Potential MRGPRX2 Involvement

Reported peak plasma concentrations for fluoroquinolones generally average 2-6 micrograms/ml after a single dose, with intravenous administration often producing higher levels than oral (174, 185–187). As seen in **Table 1**, only ciprofloxacin can activate MRGPRX2 at these levels. However, plasma concentrations can be much higher after multiple high dose administration – for example, plasma levels exceeding 10 micrograms/ml have been recorded for ciprofloxacin (188), and over 20 micrograms/ml for levofloxacin (189–191), even several hours after intravenous infusion (189). Abnormally high plasma concentrations may also occur in patients with renal impairment (192) and poor metabolism (168). Peak concentrations may be even higher, as sampling of blood during infusions usually wasn’t measured. Nonetheless, it seems likely that levels required for clinically relevant MRGPRX2 activation are only transiently achieved. This may account for the relative rarity of systemic mast cell-associated reactions, compared to other MRGPRX2 agonists like vancomycin. No measurements of drug concentrations have been made immediately after anaphylactic events that could help determine whether they might also involve MRGPRX2, nor has an analysis of allele expression been conducted.

An unusual property of many fluoroquinolones is that they accumulate in specific tissues at concentrations well above peak plasma concentrations (187, 193, 194), and can exceed those needed to activate MRGPRX2. This is linked to the lipophilicity of the molecule (194), and some are much more likely than others to distribute unevenly. The lung, especially the epithelial lining fluid (ELF), is a site of some of the highest reported concentrations (195) – for instance, levofloxacin concentrations in the ELF have been measured at over 40 micrograms/ml (189), and two hours after a single dose of moxifloxacin, ELF concentrations reached 21 micrograms/ml (196). Limited data, mostly from experimental animal models, suggest that fluoroquinolones also accumulate in cartilage (197). This may have some significance, as fluoroquinolones are associated with arthralgia, myalgia, and joint damage in some studies (198), and mast cell mediators have been shown to weaken tendons (181). In sum, this unusual tissue distribution pattern of fluoroquinolones may result in intense mast cell activation that is both delayed and restricted to certain tissues. This would not be accompanied by the typical signs of degranulation like rash and pruritus, but a closer look at mast cell, and MRGPRX2, involvement in these delayed and long-lived side effects might be justified.

## Discussion

A question that is raised repeatedly in the literature is whether MRGPRX2 activation is capable of triggering anaphylaxis. This likely would require a sustained period of high plasma drug levels, and/or expression of a rare MRGRPX2 allele with greatly enhanced sensitivity to the drug or abnormally strong signaling downstream of receptor activation. Tandem testing of MRGPRX2 allele expression, drug concentrations, and IgE titers in patients with anaphylaxis would be extremely informative; unfortunately, these tests are rarely conducted and most MRGPRX2 alleles remain uncharacterized. Ultimately, an MRGPRX2 antagonist is needed to provide direct proof of MRGPRX2 involvement. Development of antagonists is in its infancy and none has made it to clinical trials yet, though several promising candidates have been identified and rapid development on this front is expected (15).

Perhaps the most surprising finding from the analysis is how common the mild-to-moderate events are. These events are not life-threatening like anaphylaxis is, and rightly should be a lesser priority for clinicians. However, they should not be neglected, either. For instance, one allergic-type response classified as moderate is a drop in mean arterial pressure of over 20% - this certainly is not a trivial effect and may have an impact on vascular stability, especially in patients who have serious cardiovascular impairment already. Comparisons with plasma drug levels suggest that MRGPRX2 drives many of these mild-to-moderate events. An MRGPRX2 antagonist is not yet available, but if one enters clinical use, it would be interesting to see if prophylactic administration before surgical procedures lowers overall perioperative patient mortality. In sum, it is clear that there is much still to be learned about MRGPRX2 and its impact on human health.

## Author Contributions

The author confirms being the sole contributor of this work and has approved it for publication.

## Funding

This article was written with the support of the Ernest S. Bazley Trust.

## Conflict of Interest

The author declares that the research was conducted in the absence of any commercial or financial relationships that could be construed as a potential conflict of interest.

## Publisher’s Note

All claims expressed in this article are solely those of the authors and do not necessarily represent those of their affiliated organizations, or those of the publisher, the editors and the reviewers. Any product that may be evaluated in this article, or claim that may be made by its manufacturer, is not guaranteed or endorsed by the publisher.
